# Linking microarray reporters with protein functions

**DOI:** 10.1186/1471-2105-8-360

**Published:** 2007-09-26

**Authors:** Stan Gaj, Arie van Erk, Rachel IM van Haaften, Chris TA Evelo

**Affiliations:** 1 Nutrigenomics Consortium, Top Institute Food and Nutrition, Wageningen, The Netherlands; 2 BiGCaT Bioinformatics, University Maastricht, Maastricht, The Netherlands; 3 Department of Human Biology, Nutrition and Toxicology Research Institute Maastricht (NUTRIM), University Maastricht, Maastricht, The Netherlands; 4 Laboratory of Experimental & Molecular Cardiology, The Interuniversity Cardiovascular institute of the Netherlands (ICIN), University Maastricht, Maastricht, The Netherlands

## Abstract

**Background:**

The analysis of microarray experiments requires accurate and up-to-date functional annotation of the microarray reporters to optimize the interpretation of the biological processes involved. Pathway visualization tools are used to connect gene expression data with existing biological pathways by using specific database identifiers that link reporters with elements in the pathways.

**Results:**

This paper proposes a novel method that aims to improve microarray reporter annotation by BLASTing the original reporter sequences against a species-specific EMBL subset, that was derived from and crosslinked back to the highly curated UniProt database. The resulting alignments were filtered using high quality alignment criteria and further compared with the outcome of a more traditional approach, where reporter sequences were BLASTed against EnsEMBL followed by locating the corresponding protein (UniProt) entry for the high quality hits. Combining the results of both methods resulted in successful annotation of > 58% of all reporter sequences with UniProt IDs on two commercial array platforms, increasing the amount of Incyte reporters that could be coupled to Gene Ontology terms from 32.7% to 58.3% and to a local GenMAPP pathway from 9.6% to 16.7%. For Agilent, 35.3% of the total reporters are now linked towards GO nodes and 7.1% on local pathways.

**Conclusion:**

Our methods increased the annotation quality of microarray reporter sequences and allowed us to visualize more reporters using pathway visualization tools. Even in cases where the original reporter annotation showed the correct description the new identifiers often allowed improved pathway and Gene Ontology linking. These methods are freely available at http://www.bigcat.unimaas.nl/public/publications/Gaj_Annotation/.

## Background

Gene expression microarray technology plays an important role in modern biomedical research. As a result of technological innovations it is possible to measure gene expression of ten thousands of gene products at the same time. Consequently, studies using these techniques generate huge amounts of data which need to be properly analyzed. For a correct biological interpretation, knowledge of what the reporters on the array actually measure is very important. It is quite common that a substantial fraction of the used reporters are annotated with less useful descriptions like "expressed sequence tag" (EST) or "hypothetical gene". In the worst case no useful information about the reporter sequences is provided at all. To overcome this problem, annotation procedures should be directed towards finding the most informative description.

Microarray reporter sequences are either oligonucleotide reporter sequences designed using nucleotide databases such as EMBL [1]/GenBank [2], UniGene [3] andlkdjkfjd, EnsEMBL [4] or RefSeq [5], or clone sequences (cDNA arrays) that need to be sequenced before they can be annotated. Microarray developers usually provide the sequences from which the oligonucleotide sequences were derived, or the sequences on the array itself, alternatively they give the database accession number of the first BLAST-hit [6] of either of these sequences.

To improve the biological understanding of large-scale gene expression studies it is helpful to determine whether the products of the regulated genes have known biological functions or structures, or are known to be involved in metabolic or regulatory pathways. The common approach to achieve this is to visualize these genes as part of gene groups or pathways. Information needed for this kind of gene classification can be found in the Gene Ontology database [7-9], or derived from literature or protein databases. Pathways used by scientists are found for instance in KEGG (the Kyoto Encyclopedia of Genes and Genomes) [10], in GenMAPP (Gene Map Annotator and Pathway Profiler) MAPP archives [11,12] and at BioCarta [13], but such pathways are not always ideally suited for a thorough interpretation of the microarray data. For instance, KEGG pathways were constructed based on Enzyme Commission (EC) numbers [14]. It is not trivial to relate these ECs to functional proteins. But, there are cases where one single EC actually groups multiple enzyme subtypes together. For example, the human superoxide dismutase-enzyme (SOD – EC: 1.15.1.1) represents one single enzyme function in the Enzyme database. However, there are three different enzymes known with this function that are present in different parts of the cell (mitochondrial matrix [Swiss-Prot: P01479], cytoplasm [Swiss-Prot: P00441] and extracellular [Swiss-Prot: P08294]). These three SODs do not only have different cofactor binding properties but are also known to have different structures and functions. When a microarray analysis is performed at the pathway level, you do not want to associate all three enzymes with the same EC to a reaction that is actually performed by just one of them. Newer initiatives, such as Reactome, start with identifying biomolecular reactions, group them together and construct molecular pathways around them [15]. Each Reactome reaction description contains information about the proteins involved, usually through a reference to UniProt (SP). Since both signaling and metabolic pathways are understood at the level of proteins involved, it is best to annotate both the pathway node in the pathway diagram and the array reporters with the ID for the corresponding protein product. A good candidate protein database to base this annotation upon is UniProt, providing curated, high quality information about the proteins and many references towards other interesting databases, such as OMIM [16], Entrez Gene (EG) [17,18], PDB [19] and many more.

The most obvious approach towards finding a protein product for a microarray reporter sequence is by aligning the translated reporter sequence against a protein database by using the BLASTx-program [6]. This approach is used in recently published annotation tools such as TargetIdentifier [20] and GARBAN [21], that are aimed to identify protein products by improving the annotation of ESTs. Although the results of such an approach are not high in number, the quality of the hits is usually good. However, there are two main reasons why this approach is not always successful: (1) mRNA and gene sequences often contain large non-coding regions that cannot be translated into functional protein domains and therefore will not yield good high quality alignments. (2) The nucleotide sequences of clones often contain sequencing errors, leading to insertions or deletions. This will lead to frame shifts in the translated protein sequence, resulting in two or more separate (shorter) hits for parts of the sequence when using BLASTx. Of course the common way to circumvent this translation problem is to BLAST the reporter sequence against a nucleotide database. This approach is generally applied by commercial array providers [22]. It yields a maximum amount of results, but the quality of the annotation found is not always optimal: alignments can point out to something similar to the actual sequence. Finding relatively non-informative sequences, such as ESTs, and annotation results where the gene is described as "being similar to something else" is also quite common. This leads to two main problems: the quality of the hit and the quality of the annotation of the hit.

Based on the above it is clear that a good annotation method must: a) find meaningful descriptions wherever possible b) not find descriptions where the actual alignment of reporter and annotated sequence (and thus hybridization between reporter and mRNA) are not good enough c) find the best description available at the given time (i.e. a protein product); d) make use of an identifier that is commonly used in visualization and analytical tools.

**Figure 1:**
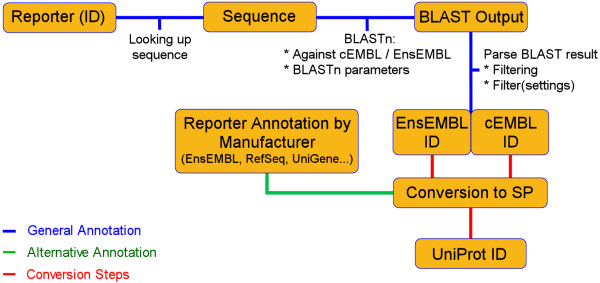
**General workflow**. When the reporter sequence is available, the annotation process starts with two separate BLAST searches. The resulting hits are filtered and then converted into UniProt IDs (SP). When only UniGene annotation is available, as is sometimes the case for self-spotted arrays based on commercial oligo or clone libraries, this annotation can be converted using specific database mining steps.

The method we describe uses a newly derived database that was created by cleaning up a redundant EMBL (cEMBL) database with the help of crosslinks within UniProt. These results were combined with a more traditional annotation method, based on BLASTn searches against EnsEMBL, followed by a conversion towards their corresponding UniProt ID(s). In a last step, both methods were combined to obtain the final annotation table (figure 1).

## Results

### Annotation results Incyte Mouse UniGene I array

**Figure 2:**
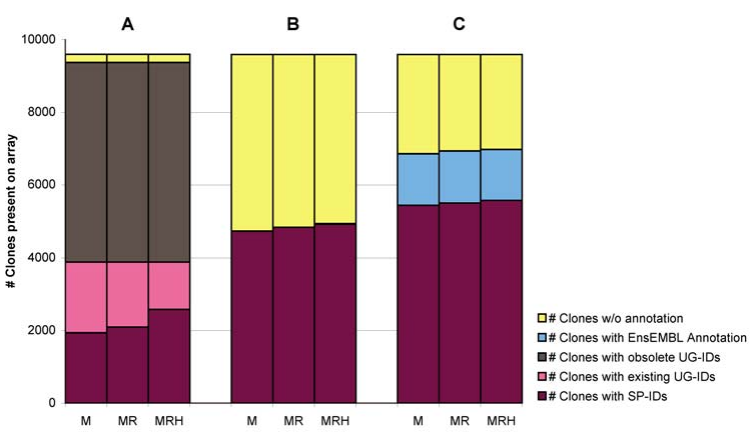
**Improving Incyte annotation**. The manufacturer provided an old UniGene annotation for 97.6% of all clones present on the Incyte Mouse UniGene I array (9,596 clones (100%)). **(A) **The first three columns show that 5,487 (57.2%) of the original clusters are no longer present in the current UniGene (UG) database. For those that are still present, UniProt (SP) crosslinks were retrieved using a UniGene specific datamining approach. From left to right: using mouse crosslinks only (M), mouse and rat crosslinks (MR) and finally mouse, rat and human crosslinks (MRH). (M: 1,939 (20.2%); M+R: 2,096 (21.8%); M+R+H: 2,582 (26.9%)); **(B) **Columns 4, 5 and 6 show the number of new annotations obtained through filtered BLASTs against the cEMBL subsets. (M: 4,736 (49.4%) ; M+R: 4,843 (50.5%); M+R+H: 4,928 (51,4%)) **(C) **Columns 7, 8 and 9 show the number of new UniProt annotations obtained through filtered BLASTs against both the cEMBL subsets and the Mouse subset of the EnsEMBL Release 41 (M: total= 6,855 (71.4%) of which 5,435 (56.6%) UniProt IDs; M+R: total = 6,931 (72.2%) of which 5,497 (57.3%) UniProt IDs; M+R+H: total = 6,973 (72.6%) of which 5,570 (58.0%) UniProt IDs).

The Incyte Mou***s***e UniGene I array consists of 9,596 clones of variable length. For annotation purposes, Incyte opted for UniGene cluster annotation for most of the clones present as shown in figure 2A. After converting the older UniGene clusters to their UniProt counterpart a large number of clusters were found to be no longer present in the mouse subset of UniGene release build 151. However, for the UniGene clusters that were still present in the current UniGene database, it was possible to annotate more than half of them with UniProt IDs. When protein similarities with rat and/or human were also taken into account, this number increased slightly.

From the total number of reporter clone sequences present 56.6% of the reporters could be coupled to a valid Mouse UniProt ID using the novel double crosslink approach, this number increased to 58.0% when references to other species were included. After combining both annotation approaches the total annotation rate ended up at 72.7%. These results are summarized in figure 2B and 2C.

A considerable number of reporters were originally annotated with less-informative descriptions (n = 5,137) such as ESTs and Riken cDNA clones (table 1). With the new approach we were able to associate 1,905 (37.1%) of these reporters with one or more proteins. When the EnsEMBL references were included as well, this percentage increased to 58.3%. In total, 2,201 reporters did not pass our high-quality criteria after being BLASTed against the primary species database, while 471 reporters aligned with more than one UniProt ID

**Table 1:** Annotation improvement of the less-informative Incyte clones

***Old Annotation***	***New Annotation***			
***Description Type***	***#Reporters***	***#Ens ID***	***#SP ID***	***% Annotated***
**Riken cDNA**	1,597	452	779	77.0%
**ESTs**	3,256	565	971	47.1%
**Hypothetical**	23	7	14	91.3%
**CDNA**	3	0	3	100%
**Gene Model**	6	1	5	100%
**Gene Trap**	7	4	1	71.4%
**Similar to**	65	16	40	86.1%
**DNA Segments**	107	22	58	74.7%
**Clones**	73	26	34	82.1%

**TOTAL**	5,137	1,903	1,093	58.3%

This table summarizes all categories of the originally less-informative descriptions on the Incyte Mouse UniGene I array after reannotation. We were able to annotate 2,795 (58.2%) of those with a UniProt identifier. In total 4,359 (90.8%) clones were given a more specific description.

SP, SwissProt/UniProt; Ens, EnsEMBL

For comparison we also annotated this array with two existing procedures. When we used the BLASTx based TargetIdentifier approach on all 9,596 Incyte array clone sequences we were able to couple 3,057 reporters with a UniProt ID, of which 2,008 Mouse UniProt IDs. Alternatively, a direct BLASTn against RefSeq was used which did find about 10% less annotations than our approach did, but was far less effective on the pathway level (see "Gene Ontology and pathway visualization").

### Annotation results Agilent G2519A Option 2 Mouse Development 44K Array

**Figure 3:**
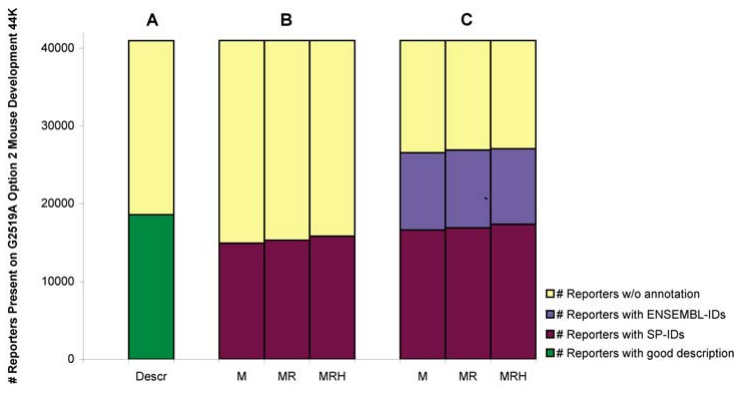
**Improving Agilent annotation**. The Agilent G2519A Option 2 Mouse Development 44K array consists of 41,013 reporters. **(A) **The annotation file contains informative descriptions for 18,609 reporters (45.4%), represented in the first column. All other reporters are non-informative, regarded as "badly annotated". **(B) **The next three columns represent the number of UniProt annotations (SP) obtained from BLASTing against the cEMBL subset(s): Mouse: 14,938 (36.4%); Mouse + Rat: 15,416 (37.3%); Mouse + Rat + Human: 15,838 (38.6%). **(C) **Columns 5, 6 and 7 show the new annotations derived from BLASTs against both cEMBL and EnsEMBL (M: total = 26,554 (64.7%) of which 16,622 (40.5%) UniProt IDs; MR: total = 26,903 (65.6%) of which 16,908 (41.2%) UniProt IDs; MRH: total = 27,077 (66.0%) of which 17,365 (42.3%) UniProt IDs).

The Agilent G2519A Option 2 Mouse Development array contains 41,013 60-mer reporters that were annotated with one or two National Institute of Aging (NIA) or National Institute of Health (NIH) Mouse Genome IDs [23]. A closer inspection revealed that less than half of the total reporter descriptions (18,609 or 45.4%) contained meaningful gene information (figure 3A). All other reporters (22,386) had a poor annotation, further illustrated in table 2. The cEMBL approach identified 36.4% of all 41,013 features with a UniProt Mouse ID. Adding the results of the cEMBL BLAST against rat and human, this number increased to 37.3% and 38.6% respectively (figure 3B). Combining both EnsEMBL and cEMBL annotation resulted properly identifying over 66.0% of all features (figure 3C). In total, 40.1% of these features were linked to one or more Mouse UniProt IDs. Adding the protein information of both rat and human species increased this number to 41.2% and 42.3% respectively.

**Table 2:** Annotation improvement of the less-informative Agilent features

***Old Annotation***	***New Annotation***			
***Description Type***	***#Reporters***	***#Ens ID***	***#SP ID***	***% Annotated***
**Riken cDNA**	9,759	2,937	2,008	50.7%
**ESTs**	369	173	132	82.7%
**Hypothetical**	348	173	52	64.7%
**CDNA**	640	325	200	82.0%
**Gene Model**	734	271	90	49.2%
**Gene Trap Library**	48	13	15	58.3%
**Intronic**	1,408	146	285	30.6%
**Similar to**	748	273	154	57.0%
**Unknowns**	7,849	1,156	1,448	33.2%
**DNA Segments**	270	110	127	87.8%
**Clones**	213	39	37	35.7%

**TOTAL**	22,386	5,616	4,548	45.4%

This table categorizes all originally less-informative feature descriptions on the Agilent G2519A Option 2 Mouse Development array (22,386) into several groups. After BLASTing their corresponding sequences against either cEMBL or EnsEMBL, we were able to relate 10,164 (45.4%) features to an improved description. For the "unknown" category more than half of the features now have an improved annotation. Of those, more than half refer to known proteins.

SP, SwissProt/UniProt; Ens, EnsEMBL

Most reporters on this array were originally associated with less-informative descriptions (n = 22,386), as shown in table 2. Our annotation methods were able to functionally annotate 20.3% of these reporters with one or more proteins. This number increased to 45.4% after adding the EnsEMBL based annotation. Our methods coupled more than one UniProt ID to 712 individual reporters.

For this array we also compared the results with a direct BLASTn against RefSeq and again our approach found about 10% more annotations.

### Comparing old versus new reporter annotations

**Figure 4:**
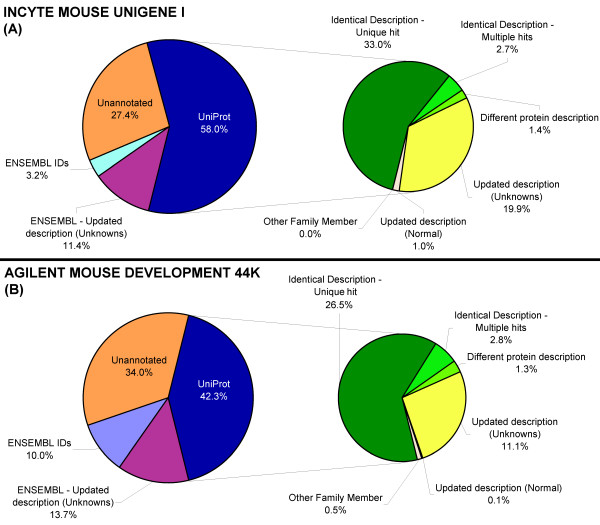
**UniGene/UniProt composition after annotation**. For the amount of reporters that were annotated with either a UniProt or a UniGene ID, only the UniProt description was compared with the description given by the array manufacturer. For the Agilent array the comparison was mainly done by comparing gene names and their synonyms. The results of this comparison are displayed in the right-half of figures A and B. The descriptions were checked if they were identical, if they belonged to a different family member, if the new annotation gave a more detailed description or if they both referred to something completely different. Reporters that were confirmed were also screened for specificity (i.e. specific for that gene or not). **(A) **For Incyte we were able to identify 72.6% of all features. A very small part of the clones (1.4%) did not correspond with the new annotations. **(B) **For Agilent, a large number of features associated with a protein ID correspond with the Agilent Annotation (26.5%), while 1.3% were annotated as different.

For most of the array reporters both original and newly found descriptions were identical, but some reporters were annotated with either several functionally different proteins or with different protein family-members. In some cases the new annotation updated the given description. For example, "RAS RELATED PROTEIN RAB" and "NEUROPILIN AND TOLLOID LIKE" became respectively "Ras-related protein Rab-6B" and "Neuropilin-2 precursor (Vascular endothelial cell growth factor 165 receptor 2)". The outcome of this comparison is for both arrays illustrated in figures 4A and 4B.

### Comparing our cEMBL versus RefSeq/TargetIdentifier annotations

To compare the results of each method (RefSeq, TargetIdentifier) with our cEMBL approach, 1,000 reporters were randomly selected for each array using the C++ random number generator. When both methods yielded a protein hit, the reporter annotations were compared based on a match in either the gene name or in the protein description (see additional file 1).

For over 90% of the array reporters that yielded a protein product using both the cEMBL and the RefSeq approach the same protein was found. For some of the conflicting annotations our approach yielded more than one UniProt ID and the corresponding one could be found lower in the list (Agilent: 3; Incyte: 7). Another part of these reporters linked to a different family member (Agilent: 9; Incyte: 9), whereas a small part of these array reporters did not overlap at all (Agilent: 7; Incyte: 10). One conflicting reporter on the Agilent array (A_66_P102868) was flagged as really bad since our approach found more than ten protein annotations and they did not correspond to the RefSeq protein.

For TargetIdentifier the overlap was somewhat smaller (83%). This was to be expected since TargetIdentifier aligns the sequences against more species than only our species of interest.

In general, our cEMBL method was able to find more meaningful hits (i.e. proteins) compared to the other approaches. This is reflected by the number of reporters that were accepted in the GenMAPP gene database (see Additional File 1).

### Gene Ontology and pathway visualization (Table 3)

**Table 3:** Gene Ontology classification and pathway visualization using all approaches

	**On Array**	**Annotated**	**In Pathway Gene Database**	**On Local Pathways**	**On GO Levels**
**Incyte UniGene I**					

**Old**	9,596	9,370	3,730	924	3,130
**TargetIdentifier**	9,596	2,008	1,990	615	1,827
**RefSeq**	9,596	6,359	4,356	1,110	3,777
**Our Approach**	9,596	6,973	6,710	1,609	5,597

**Agilent G2519A**					

**Old**	41,013	17,325	17,041	2,351	10,554
**RefSeq**	41,013	25,625	15,763	2,286	10,408
**Our Approach**	41,013	27,077	25,694	2,927	14,482

This table summarizes for each approach all reporters that could be imported and visualized using the GenMAPP program. A clear distinction has been made between the local MAPPs released on September 20^th^, 2006 and the Gene Ontology database, built in the MAPPFinder program of GenMAPP (Release date: August 25^th^, 2005). In general, improving the annotation yields better pathway visualization.

For the Incyte array (n = 9,596), the GenMAPP 2.1 program [11,24] was able to link 5,287 newly annotated reporters to unique GO nodes, compared to 3,130 reporters based on the original UniGene annotation. Linking these reporters with 101 local mouse pathways included in the GenMAPP program, it was able to associate 3,730 old UniGene IDs with unique genes present in their local database. Out of this amount, only 924 genes could be shown in a local pathway. These numbers increased after applying our annotation methods to respectively 6,710 and 1,609, whereas the RefSeq approach ended up with 4,356 proteins accepted in the gene database of which only 1,110 could be found on a local pathway.

For the original Agilent array annotation (n = 41,013) it was possible to extract and convert 17,325 reporters towards a usable RefSeq Protein (NP) Identifier. After applying our methods, there was not only a higher number of unique genes accepted in the GenMAPP database, but the amount of reporters visualized on GO level increased from 10,554 to 14,473 as well. Shifting our focus towards the local pathway level, our methods added 576 extra reporters that now can be linked to a gene present in a local pathway. The RefSeq approach found only about 10% fewer gene annotations (25,625) when compared to our approach. Converting these DNA IDs (NM/NX/XM/XR) to their protein counterparts yielded 20,322 protein (NP/XP) annotations. Only 15,763 of these RefSeq protein IDs were accepted in the GenMAPP mouse gene database, visualizing 2,286 genes on a local pathway and providing 10,408 Gene Ontology classifications.

### Improving pathway visualization

#### a) Classification of the Incyte array reporters (Table 4)

**Table 4:** Linked reporters to the GenMAPP gene database

		**Incyte **cEMBL		**Agilent **cEMBL	
**(A)**		**YES**	**NO**	**YES**	**NO**

Old	**YES**	3,037	814	15,018	2,023
	**NO**	3,659	2,086	10,676	13,296

**(B)**		**YES**	**NO**	**YES**	**NO**

RS	**YES**	4,002	320	14,601	1,162
	**NO**	2,694	2,580	11,093	14,157

These tables display the total number of individual reporters that were either accepted or rejected in the mouse gene database by importing both the original (**Old**), the RefSeq (**RS**) and our combined (**cEMBL**) annotation. (A) Comparing the original annotation with our combined annotation approach; (B) Comparing the RefSeq-based annotation with our combined annotation approach;

For the Incyte array we successfully increased the reporters linked to unique genes in the gene database from 3,730 to 6,710. Surprisingly, 814 Incyte reporters could originally be visualized using the old UniGene annotation, but after applying our combined approaches they did not appear on any MAPP. Of those 814 reporters: (a) 724 reporters remained un-annotated. Re-evaluating the BLAST results of these reporters showed that about half of them (384) showed some alignment with a gene sequence (> 70% alignment), but these alignments failed our quality criteria. (b) 40 reporters were associated with either a rat or human UniProt ID and 15 with a rat or human EnsEMBL ID that could not be converted to UniProt. Because the GenMAPP MAPPs and gene database are species-specific, it was not possible to visualize the human and rat based annotations. (c) The remaining 35 reporters were linked to a mouse identifier that was not present in the mouse gene database.

#### b) Classification of the Agilent reporters (Table 4)

Similarly for the Agilent array the amount of reporters linked to unique genes in the GenMAPP gene database increased from 15,018 to 25,694. After applying our new annotation methods, about 2,023 probes could not be linked to pathway nodes again. For these 2,203 probes: a) 1,524 probes remained un-annotated. Of those only 84 showed an alignment of > 70% with a gene sequence while the others only gave very poor hits. b) 375 probes referenced in our new annotation to a rat or ruman identifier. C) 124 probes were linked to Mouse UniProt IDs that were not available in the GenMAPP mouse gene database.

#### c) Local pathway improvement evaluation using GenMAPP

**Figure 5:**
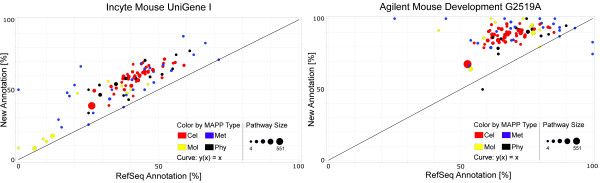
**GenMAPP local pathway comparison**. This plot shows for each local pathway in GenMAPP the percentage of total genes in the pathway that we were able to visualize using our approach (y-axis) versus the RefSeq-based annotation (x-axis). Each dot in the plot represents a single pathway, colored by its main category (blue: Metabolic Pathways; red: Cellular Pathways; yellow: Molecular Pathways; black: Physiological Pathways). The sizes of the dots represent the size of the map, varying between 4 and 551 gene product identifiers. For the Incyte array (left) we were able to improve the visualization for 96 out of 101 pathways. Three pathways remained identical. Our methods were able to add two more pathways to the list. For the Agilent array (right) 92 pathways showed an improvement in visualization power, whereas 3 pathways remained unchanged and 4 small pathway decreased in visualization power. Note that 10 small pathways (pathways with less than 20 genes present) are now fully visualized due to our efforts.

The improvement of the percentages of genes that can be visualized in individual pathways is shown in figure 5. Using our annotation methods, the number of gene products that can be visualized increased on 96 (Incyte) and 92 (Agilent) out of 99 pathways compared to what could be achieved with the RefSeq-based annotation (see additional file 2).

**Figure 6:**
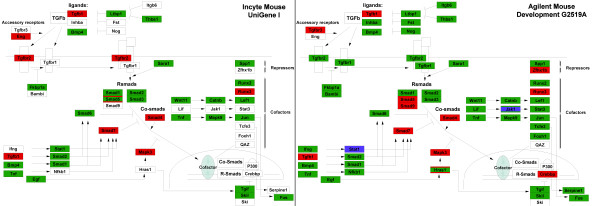
**Improvement of local pathways in GenMAPP**. This figure represent a Transcription Growth Factor Beta (TGF-B) signaling pathway that was improved after applying our new annotation methods for both the Incyte (left) and Agilent (right) arrays. Genes visualized using the RefSeq annotation could again be visualized with the new annotation (green box), whereas the new annotation added a large amount of genes that can be used for visualization purposes (red box). Additionally, the RefSeq approach found some genes which our approach was unable to find (purple box). Boxes with more than one color indicate that that gene is covered on the array by multiple reporters.

In general, our methods largely increased the amount of genes visualized on a number of important pathways. To illustrate this improvement, one pathway is shown in figure 6 where the newly mapped gene products are shown in red, while the gene products that could be visualized using both the RefSeq and our new annotations are colored green. The blue boxes represent genes that were only found using the direct RefSeq BLASTn approach.

## Discussion

When performing microarray experiments, where the expression of tens of thousands individual genes is measured simultaneously, it is important to correctly understand the biological outcome. To achieve this, array reporters need to be annotated with the gene or gene product that they correspond with. Nowadays, array producers provide annotation files containing necessary information for their arrays, such as the database identifier of the gene of which the reporter was based on, the functional name of the gene (usually derived from the database identifier) and/or the actual reporter sequence. The quality of these annotation files can differ between array producers. But even if the annotation quality is satisfactory at the moment of purchase, it is important to keep the annotation up to date. Gene expression data repositories like GEO (Gene Expression Omnibus) [25] and ArrayExpress [26] contain more and more experimental data. An important usage option for these repositories is to do future integrated analysis, combining different kinds of data. Before this can be done, it is crucial to have adequate and up-to-date annotations. If these annotations are not updated while genome sequence databases are, we do not only lose part of the benefit of the improvements of genome annotations, but we will also lose information since older annotations do not necessarily couple to the data used in newer analytical tools. This problem often occurs when the reporters are based on UniGene IDs. For the Incyte array used in this paper originally 97.6% of the reporters were annotated with a UniGene ID. A large fraction (57.2%) of this annotation has become useless for pathway mapping by now since the cluster IDs do not longer exist in the database. This implies that it is important that databases keep track of older IDs that have been used like, for instance, EnsEMBL and UniGene do, although UniGene does not archive its obsolete cluster sequences.

Nowadays, many annotation tools are available that aim to functionally annotate a given reporter ID with a specific Gene Ontology (GO) classification [21,27-29]. A complementary approach is to interpret the data in a biological way by making use of pathway visualization tools, such as GenMAPP [11,24], MetaCore [30] and/or ArrayXPath II [31]. These tools link an element in a pathway with a specific identifier, usually by an EnsEMBL, UniProt, UniGene, Entrez Gene or GO ID. Hence, to allow the biological interpretation in a pathway context, the goal should be to derive descriptions that contain the physiological most useful information for each reporter, i.e. an identifier of a protein or other gene product. Some annotation methods use a BLASTx approach to find protein descriptions [20,21,32]. Other annotation tools do not include a BLAST search in their annotation procedure, but basically try to link entries from different databases to each other using a reference ID [33-35]. A few examples of these tools are the Database for Annotation, Visualization and Integrated Discovery (DAVID) [33] and the Annotation Builder Library (AnnBuilder) present in the BioConductor module of R [36,37], where the user has to supply a list of specific gene identifiers which will then be further linked towards other databases. If the gene identifier involved is correctly coupled to the array reporter, then such tools will allow us to find database reference IDs in numerous genomic databases. If the gene identifier is not correctly coupled, then the biological interpretation will go astray [38]. Thus, for microarray experiments it is important to start from the beginning, i.e. by BLASTing the reporter sequence used on the array. During the last years much effort has been put in redefining the annotation of the Affymetrix GeneChip arrays [39,40]. A more recent paper by Harbig et al. describes how they re-BLASTed all Affymetrix Human Genome U133A 2.0 target sequences and compared them with the annotation provided by Affymetrix. Their findings concluded that even for this often used chip about one third of the probe sets could be updated [41]. Furthermore, to facilitate biological understanding it is imperative to continually keep the annotation up-to-date and as accurate as possible, even if that means annotating a single reporter with more than one gene. Affymetrix is aware of this problem and use an "x_at"-tag at the end of the probeset name to indicate that that probeset can detect more than one gene. But these x_at probe sets are always described with only one gene product and the other products are thus not normally included in the analysis. A related problem exists when pathway visualization tools are used. These tools do not offer a solution yet on how to visualize reporters that will hybridize strongly with more than one gene target. When researchers do know all genes that could be detected by a specific reporter, they could validate these results by PCR-based techniques using all this information. Our method therefore aims to offer the end-user as much information as possible about reporter specificity: for reporters that detect more than one gene or protein product, the description, gene name(s) and identifiers are given for all possible near-perfect alignments. It is then up to the end-user to decide if they want to include the reporter in their further analysis procedure.

Because of previous experiments performed on both the Incyte UniGene Mouse I and the Agilent G2519A Option 2 Mouse Development 44K arrays we wanted to reanalyze the data and use existing pathway tools to see if more biological information could be extracted. The existing array annotations for both arrays were problematic: (a) the Incyte array contained many annotations relating to UniGene clusters that have been removed from the UniGene database and are therefore no longer useful; (b) the Agilent annotation was based on the NIA mouse cDNA Project [23]. The 41,013 reporters present on this array were derived from genomic mouse ESTs and clones, explaining the non-informative description for a large amount of the reporters present (54.6%).

Because existing annotation methods using the BLASTn algorithm [38,42,43] did not fulfill our criteria, we decided to develop a new method that followed a few strict criteria. UniProt was chosen as reference database because its entries are crosslinked to other databases that give relevant information for further analysis. Filtering the nucleotide EMBL database, by only holding on to references from within a species-specific UniProt subset, resulted in a less redundant and more meaningful cleaned EMBL (cEMBL) subset. A direct BLASTn against this cEMBL yields hits that are directly related to protein information. Additionally, we performed a BLASTn against the transcripts present in the EnsEMBL database and crosslinked the EnsEMBL ID back to end up with a UniProt ID.

The EnsEMBL project plays an important role in current bioinformatics related to microarray annotation. It contains much information from genomic sequence up to the protein level. Identification of the relation between a microarray reporter and an EnsEMBL gene also allows evaluation of other aspects described or referenced in EnsEMBL like gene splicing, variants, SNPs and gene orthologs. Nowadays, there are many tools available that accept EnsEMBL IDs. Thus, if an EnsEMBL ID contains no crosslink to UniProt, that ID can still be used for continued analysis. This powerful feature makes EnsEMBL an ideal candidate database to BLAST against. Traditionally annotation tools often used UniGene as reference database [38,43]. This is in principle not wrong, but we strongly discourage this approach since it is known that after each UniGene update clusters are split, joined together or removed from the database, often resulting in obsolete annotation for that reporter gene. Additionally, the extra information coming from within UniGene is more limited in situations where the complete genome of an organism has already been sequenced. UniGene based approaches, as described above for the older Incyte annotations, are still useful for reporters that were historically annotated with UniGene clusters and for species where EnsEMBL builds are not yet available. Many labs still have clone collections for which they only have UniGene annotations and no sequence information.

After applying our annotation methods the number of reporters annotated with a UniProt or an EnsEMBL ID on either array was over 60%. This is quite high, considering the large number of reporters that originally had less-informative descriptions (tables 1 and 2). Whenever the original annotation also lead to a meaningful description the content of the original and our new annotation was most often identical, although our methods updated the description of 1.0% (Incyte) and 0.1% (Agilent) of these reporters for instance to a specific protein family member. For the Incyte array a lot of UniGene cluster IDs did no longer exist, preventing further analysis relative to GO terms and pathway mapping, even if the descriptions of the lost clusters was in principle correct. This loss of UniGene clusters can be explained by both an improvement in the clustering algorithms used and an increase in the amounts of sequence data processed. Furthermore, depositors can withdraw poor sequence data at a later time, resulting in unresolved sequences. The old Incyte annotation did give annotations for a number of reporters (7.3%) that our new methods failed to annotate. This is primarily due to the higher alignment criteria that we applied. If these alignment criteria would be lowered, then the amount of reporters annotated would increase (but one would have to verify that the corresponding sequences actually do hybridize). For Agilent, the number of probes that our methods failed to reannotate amounted to 4.9% of the total. For about one third of these lost Agilent annotations the original description was non-informative ("Riken cDNA Clone", "Similar To", "EST"). The rest originally gave specific for gene products, but could not be confirmed by our annotation methods. The cEMBL database contributed a considerable amount of direct protein annotations for both arrays. Since thus derived annotations are more trustworthy than the ones built on the EnsEMBL approach we preferably kept them whenever the two approaches yielded different UniProt results.

We compared the results of our approach with existing approaches. First we looked at TargetIdentifier [20]. This web service attempts to identify ESTs as full-length cDNAs and to provide further functional annotation. First, the EST sequences are divided into categories, based on the presence and location of a start- and stop-codon and passed through a BLASTx algorithm. This approach circumvents part of frame shift problems in a general BLASTx approach that were mentioned in the introduction as the position of start- and stop codon allows for a choice of a fixed frame. Where our approach found 6,973 annotations for the Incyte array, TargetIdentifier found only 3,057 UniProt ID's and only 2,008 of these were from the correct species. We also used a direct BLAST against RefSeq with the standard (NCBI) E-value of 0.01 and the same filtering for at least 90% alignment that we used in all other BLAST procedures. The RefSeq approach improved the original annotation of the Incyte array, but was less successful on the Agilent Mouse Development 44K array. Our methods found about 10% extra annotation on both arrays compared to the RefSeq approach (see table 3). A much larger difference occurred when we looked for usability of this annotation in pathway mapping and Gene Ontology classification (see below). This is caused by the fact that GenMAPP uses an EnsEMBL based gene database. RefSeq protein IDs (and any other type of ID) are only accepted if they are linked to EnsEMBL genes in the EnsEMBL database itself. EnsEMBL only includes the link between nucleotide and protein identifiers when this identity is confirmed. For most (90%) of the protein identifiers linked to RefSeq NM IDs that are not found in EnsEMBL this is not the case (they are given XP IDs not NPs). The remaining 10% is probably caused by the RefSeq and EnsEMBL builds being out of sync (RefSeq being newer). To verify that the EnsEMBL gene database is the cause of the lower acceptance of RefSeq IDs we used BioMART on build 41 of the Mus Musculus EnsEMBL database to associate the RefSeq DNA IDs, resulting from blasting against RefSeq, with NP (protein) ID's. For both the Agilent and Incyte reporters the number of RefSeq NP ID's found in this way corresponded exactly with the number of genes accepted in the GenMAPP gene database starting from the larger numbers of NP identifiers found in RefSeq through conversion in RefSeq itself. (Incyte: 4,356; Agilent: 15,763). The background of this problem is that we understand biological pathways as protein functionality, and the proteins that are part of a pathway are primarily described by this protein functionality (through the pathway backpages) while array reporters are normally annotated with nucleotide products. The mapping of proteins to corresponding genes in databases like RefSeq and EnsEMBL is still generally better than the mapping of nucleotide sequences to proteins.

We tried to evaluate whether the increase in coverage comes from a decrease in specificity, meaning we annotated reporters that should not be annotated, or that we coupled the original nucleotide annotation to a protein where that is not correct. Using our alignment quality criteria the actual thermodynamic hybridization conditions correspond to a one basepair mismatch in a 25 base sequence. This essentially is what is used for the Affymetrix match/mismatch method and is known to lower hybrizations to a large extend (we are aware of problems with the mismatch usage on Affymetrix, but that is not relevant here). As a result, false positives (sequences annotated with something they do not really hybridize to) are less likely than they are in approaches where the first BLAST hit is used with only an E-value cut-off. Database crosslinks from nucleotide databases to protein databases are often created automatically. The crosslinks from protein databases (especially the SwissProt part of UniProt) are highly curated and errors are less likely. Since the initial step in our procedure uses the crosslinks from UniProt to EMBL it makes sense that fewer false positives will occur. To evaluate this further we compared the outcome of the annotation for 1,000 reporters for our approach and the RefSeq and TargetIdentifier approaches in more detail (extra tables 1 and 2 in additional file 1). In general, when protein descriptions for the alternative annotations are compared they are identical. The most important differences are: 1) Compared to the TargetIdentifier approach both our method and the RefSeq method found many more results. This is due to the intrinsic limitations of the TargetIdentifier approach which is based on BLASTx. Also the TargetIdentifier approach sometimes finds hits for the wrong species which cannot be used in pathway analysis. 2) Our approach was able to find protein descriptions where the RefSeq approach found only corresponding nucleotide sequences for 12–14% of the reporters. 3) The other way around, the RefSeq approach found 8–10% proteins that we only found as nucleotides. Most of these could in fact be mapped to the GenMAPP gene database as well using the nucleotide descriptions. 4) We found 5–10% extra protein descriptions where the RefSeq approach found nothing. This is the most suspect group for possible false positives. For this group we checked individual reporter descriptions for the hits that we found in cEMBL database. These were shown to indeed contain a small number (3 out of the 1,000) of possible false positives as they pointed to shorter (< 10K) BAC clones. These clones were included in the cEMBL database since they are referred from UniProt, but the actual hit might occur away from the relevant gene sequence.

While comparing the old and the new annotations we encountered some situations where a microarray reporter sequence corresponded with more than one protein: for example, Agilent reporters A_66_P115710 and A_66_P110436 resulted in finding high quality alignment against several genes. After filtering, both reporters yielded different unique UniProt IDs. For A_66_P115710 they were respectively "Histone H2A type 1" [Swiss-Prot: P22752], "Histone H2B type 1-B (h2B-143)" [Swiss-Prot: Q64475] and three other family members, while for A_66_P110436 they were "Ankyrin repeat domain-containing protein 40" [Swiss-Prot: Q5SUE8] and "Cisplatin resistance-associated overexpressed protein" [Swiss-Prot: Q5SUF2]. In both cases the conclusion would be that the specificity of the reporters' sequence is low, allowing it to detect either more than one family member or even two functionally very different proteins. In the latter case, the reporter should be omitted from further analysis. In some specific situation both EnsEMBL and cEMBL found more than 5 different UniProt IDs for 81 Agilent reporters (e.g. A_66_P135394, A_66_P139383 and A_66_P131399). Furthermore, the lists of proteins found showed no overlap for these reporters. Direct sequence analysis and evaluation of the annotations indicated that these reporter sequences were originally unknown or derived from Riken cDNA. For these 81 Agilent probes, it is clear their annotation should be omitted from further analysis. This demonstrates that, next to finding the best possible description, reBLASTing the sequence adds an extra quality check for the reporter sequence used.

After the reporter sequences are annotated, pathway visualization tools can be used to increase understanding of the biological outcome such as the GenMAPP program. The original UniGene ID's of the Incyte array could be used directly for GenMAPP analysis, but this off course yielded poor results since many of those UniGene ID's are outdated. For the Agilent array we verified whether the old annotations could still provide useful results by the use of methods that would couple them to Ontology terms: a) by using FatiGO [29], since the documentation explicitly claims that that webservice accepts Agilent Feature IDs. This approach was unsuccessful, because no single reporter could be linked to a GO term. b) by using the EnsEMBL database, that contains crosslinks towards Agilent reporters. In the end, this approach was not feasible, since the probe associations stored in EnsEMBL belong to a different array type (Agilent Mouse Whole Genome). We concluded that these methods were unable to answer our question, so a work-around was created that visualized these genes in GenMAPP (and thus in GO) by converting the RefSeq DNA Identifiers directly to their RefSeq Protein counterparts. For only ~17.000 of the ~25,000 Agilent reporters that contained a RefSeq reference in their original annotation were we able to find a RefSeq protein (table 3). This was not very different from the numbers we got when we did a direct BLASTn against RefSeq for comparison.

Using MAPPFinder, the results of our combined annotation approaches increased the amount of visualized gene products in both the local pathways and in the Gene Ontology classification. For both arrays, there were a few exceptions where the RefSeq approach was able to visualize some genes which our approach could not. The RefSeq approach found 1,162 annotations that we did not find while we found 11,093 reporters in the gene database that the RefSeq approach did not find (see table 4). A manual reblast of these sequences [e.g. Agilent: A_66_P12856] against both databases indicated that the difference is mainly due to differences between the cDNA sequences present in RefSeq and EnsEMBL: i.e. perfect hits against RefSeq versus no hit at all in EnsEMBL. Apart from this, the improvement in the amount of gene products mapped was large: the number of genes that could be mapped to a local pathway increased for Incyte from 924 (old) to 1,110 (RefSeq) to 1,609 (new). For the Agilent array a BLASTn against RefSeq found fewer sequences than the original Agilent annotation, probably because of the extra alignment criteria applied. As a result it was possible to visualize a slightly smaller amount (2,286) of genes in the local pathways, compared to the original (2,351), and substantially less (2,937) than found with our approach. Even with these relatively small changes in numbers of annotated genes the visualization power increased for a quarter of all pathways when comparing the RefSeq approach to the original annotation, but decreased in about half of all pathways. This increase was even stronger for the larger pathways. A full list of these individual pathway rankings are available as additional file 2 or can be downloaded from our servers [44].

## Conclusion

The usage of a high quality check for each individual reporter annotation, by requesting > 90% alignment identify, ensures that with the method described only those gene products are included in the annotation that will really hybridize. In general, the methods described in this paper increased the quality of the reporter annotations and increased the amount of annotated reporter sequences that can be visualized using pathway visualization tools.

## Methods

All methods were scripted using Perl. The novel method searches for a high quality alignment against a cleaned-up EMBL nucleotide database (cEMBL), only containing nucleotide sequences referenced from UniProt and for which we included a crosslink to the referenced UniProt entry if necessary. The second method is based on a more traditional approach where reporter sequences are first identified using sequence alignment based searches in a nucleotide database, such as UniGene or the one present in EnsEMBL. Protein identifiers are then added based on crosslinks present in the database searched. We used cDNA sequences in the state of the art EnsEMBL database for this purpose. These two approaches were applied to two different commercial arrays.

The scripts can be executed in both Windows and UNIX environments, using local installations of the Perl Interpreter [45] and the NCBI BLAST program [46] and can be run species-independently. Both Perl-scripts and the annotation manual are publicly available [47,48] (Category: Annotation Tools). All databases used for these procedures can be requested from Stan Gaj.

### Microarrays and reporter sequences

For this study we reannotated an old Incyte Mouse cDNA UniGene I array (9,596 reporters) and a more recent Agilent G2519A Option 2 Mouse Development 44K (41,013 reporters). Before our methods were applied, it was imperative to obtain the sequences belonging to all microarray reporters in FASTA format. For Incyte, clone sequence IDs were directly obtained from Incyte Genomics, Inc (Palo Alto, CA) and their sequences were downloaded in batches of 3,000 sequences by looking up these IDs in the Incyte Gold database. Alternatively, the Entrez Gene identifiers could have been used to retrieve the clone sequences out of the Entrez Gene database (formerly known as LocusLink) [49]. For the Agilent array, the 60-mer feature sequences were requested from the Agilent website [50].

### General workflow

The general workflow used is illustrated in figure 1. After the reporter sequences were obtained, a BLASTn against an approach-specific database was initiated. These databases were prepared to BLAST against using the NCBI formatdb program, part of the BLAST distribution. In BLAST it is possible to influence the calculation of the bit-score assigned to each aligned sequence by changing several parameters. To prevent spurious hits, the maximum expectation value (E-Value) was decreased from 10 to 10^-6. ^This E-value is dependent on the length of the query sequence, length of the aligned sequence and the length of the database BLASTed against. Lowering this value was possible because the size of the database sequences was large enough to find significant hits at that level. The gap-opening penalty (GOP) and gap-extension penalty (GEP) were both set to 1, lowering the penalties for irrelevant sequencing errors that often lead to single base insertions or deletions in groups of equal bases. To increase the sensitivity of the search, the word size was decreased to 8 (default: 11).

The generated BLAST report file was parsed and filtered by criteria that allow selection of only very high quality alignments: the reporter sequences must (a) contain a minimum length of 40 bp (on the Incyte array 4 clone sequences were actually shorter than 40 bp, meaning we did not even try to annotate those), and (b) have a total alignment of more than 90% of the length the reporter sequence. Some alignments contain more than one short fragment hit with the same database sequence. Our filter keeps track of such occurrences and checks if there is no overlap between the fragments before adding them up and applying the filter criteria.

The 90% match threshold is based on the melting temperature T_m _of the probe-target hybridization on the array. The melting temperature T_m _can be approximated by the following formula:

T_m _= 81.5 + 16.6(log_10 _[MC]) + 0.41(%GC) - B/N

[51,52]

[MC] molar concentration of monovalent cations

(%GC) percentage of G and C in the chain

N the chain length

B constant = 600 (some publications state B = 675 for chains < 100 nucleotides)

Assuming that the mismatches effectively shorten the chain length and keeping the other parameters constant, the ΔT_m _can be estimated between two hybridizations A and B as:

ΔT_m _= T_m, A _- T_m, B _= -B/N_A _+ B/N_B_.

We tried to keep the ΔT_m _in the order of magnitude of those used by Affymetrix. On this platform there are perfect match (PM) probes of 25 nucleotides and mismatch (MM) probes of the same length but with one nucleotide (the 13th) changed to its complement.

In the case of an Affymetrix probe set, the formula would be:

ΔT_m _= -600/25 + 600/24 = 1

For the 60-mer two-color probes, we get for a 90% threshold match:

ΔT_m _= -600/60 + 600/54 = 1.11 ≈ 1

After passing these filters, IDs from the queried database were associated with every individual reporter sequence. In a final step, these new identifiers were converted into a UniProt ID, and combined into one annotation file. All information about the used databases is described in table 5.

**Table 5:** Used databases

***Name***	***Version***	***File(s) used***	***Download Location***
**EMBL**	88.00	Rel_std_mus01.dat ... Rel_std_mus05.dat	
**UniProt**	51.00	uniprot_sprot.dat reldate.txt	
**EnsEMBL**	41.00	Mus_Musculus....cdna.all.fa Rattus_Norvegicus....cdna.all.fa Homo_Sapiens....cdna.all.fa	
**RefSeq**	20.00	mouse.rna.fna.gz	

### cEMBL: Cleaning up EMBL

The curated information present in UniProt was indirectly used to clean up a redundant, species-specific subset of the EMBL database. First, the SwissProt part of the UniProt database was split in multiple species-specific subsets. Next, UniProt entries in each species-specific subset were mined for cross-references towards EMBL. If a UniProt entry contained one or more valid crosslinks to EMBL, the corresponding entry in EMBL was copied into the new cleaned EMBL (cEMBL) database. A few quality criteria were included to assure that cEMBL consisted of only high-quality sequences: (a) each UniProt crosslink within an EMBL entry must point back to the UniProt entry that made us find it. If such a crosslink back did not exist, or if the crosslink did not point to the correct UniProt entry, a new database field containing the crosslink information was added to the cEMBL entry, resulting in bidirectional crosslinking between UniProt and cEMBL. All this crosslink information was saved in a tab-delimited text-file (b) UniProt entries sometimes contain an EMBL crosslink to large genomic DNA sequences. Such sequences are less useful to identify reporter sequences because of splicing and the presence of small repeats, resulting in smaller or overlapping hits. Large genomic DNA sequences contain a considerate amount of coding sequences, allowing more than one protein to be found in that given sequence. To avoid these problems, the genomic DNA sequences were automatically removed from cEMBL, based on sequence length (> 10,000 bp) and the phrase "genomic DNA" in the ID field. In numbers, the EMBL mouse database (release 88) consisted of 195,693 individual entries of which 32,470 contained a crosslink from UniProt. From this number our extra genomic cDNA criteria filtered another 1,245 entries, ending up with a mouse-specific cEMBL subset of 31,225 entries with existing or added links to protein entries in UniProt.

With the cEMBL database and the crosslink table in place, array reporters were evaluated using a BLASTn search against the cleaned subset of EMBL. After parsing the BLAST-output and applying the quality criteria mentioned above, the cEMBL IDs found were converted into a UniProt ID using the crosslink table.

Some reporter sequences did not yield any good hits after the first run; those sequences were reBLASTed against a cEMBL subset of a related species. This was done for the mouse arrays used in this study in the following order: mouse, rat and human. This order would be different for microarrays targeted to other species.

### EnsEMBL: A more traditional approach

Like in many common annotation procedures we tried to align the reporter sequences with EnsEMBL gene identifiers. For this approach, a local cDNA FASTA copy of the EnsEMBL Mouse Release (build 41) was required. After a BLASTn search against this database, the high quality alignments were extracted from the BLAST report file using our filter-settings as defined above.

The conversion table for this approach can be generated in two possible ways: a) by downloading the UniProt (UniProtKB/SwissProt) database and extract all crosslinks towards EnsEMBL; b) By using EnsEMBL's online BioMART toolset to extract all references in EnsEMBL. This paper used crosslinks from within UniProt (generated by the cEMBL approach), depending on the higher curation level present in the UniProt database. We opted for the first approach, even though BioMART is able to convert more EnsEMBL IDs towards UniProt. These extra IDs are mainly references to the SPTrEMBL part of the UniProt database. Since that would also include hypothetical proteins, we chose for the more stringent approach starting from UniProt.

The low quality alignments that did not yield an EnsEMBL ID were reBLASTed against the EnsEMBL Rat cDNA subset, followed by the EnsEMBL Human cDNA subset if they did not yield any result in the rat subset.

### Comparing annotations

Each approach resulted in a filtered list, containing the BLASTed reporter ID and their corresponding UniProt or EnsEMBL IDs. To extract as much biological information as possible, these lists need to be combined and screened for identical or different hits. For screening purposes, a flag has been implemented for each reporter, indicating the quality of the annotation. If the IDs found by both the cEMBL and the classical approach were not identical, then we continued with the cEMBL UniProt ID, unless the cEMBL UniProt ID was not related to the primary species (here: mouse) while the other one was. This choice was based on the quality of the databases used: the cEMBL database is based on curated information present in both SwissProt and PIR, now combined in UniProt while the EnsEMBL database is created by automated processes.

For the Incyte arrays, the description for each reporter, provided by the array manufacturer, was compared with the description of the UniProt annotation obtained from the methods described above. This was done using MS-Excel and only for reporters that yielded a valid UniProt ID. This was much easier for the Agilent Mouse Development array, since most reporter in the Agilent annotation file (last release dated 06-01-2005) were annotated with a gene name. String identity of these gene names and the ones derived from UniProt was evaluated using a simple Perl script. When string differences occurred, further evaluation was done manually. If a reporter gene name corresponded with one or more protein products, this was counted as a match.

### Multiple-species annotation

For some species it is possible to annotate a reporter with a protein counterpart of another species closely related to it. There are genes that are present in other species as well, containing an almost identical gene sequence. This is the case for Mus Musculus (mouse), Rattus Norvegicus (rat) and Homo Sapiens (human). The scripts were adjusted to find annotations based on to these related species in case no native hits were found. We only searched for a rat or human annotation when no high quality alignment was found for the mouse.

### TargetIdentifier

To illustrate the results of an approach that implemented a BLASTx algorithm, we used the TargetIdentifier web service [20]. This service tries to annotate unknown sequences and ESTs with a protein product (UniProt). One drawback of this online web tool was the ability to submit a maximum of 1,000 sequences for each run. Therefore the reporter sequences of only the Incyte array were used to test out this approach. These reporter sequences were split into separate files containing 1,000 sequences each and were uploaded on the TargetIdentifier website. The default criteria were kept (BLAST E-value cut-off: 10^-5 ^or less and maximum list ranking of 5).

### RefSeq: Using a similar non-redundant approach

For comparison we used the mouse specific part of the non-redundant curated RefSeq database (release 20) to BLAST our reporter sequences against with the default BLAST parameters. The resulting hits were selected when they met our quality alignment criteria (> 90%).

The BLAST database was downloaded from NCBI and prepared using the NCBI formatdb program (table 5). After filtering out the hits, we converted the high quality alignment RefSeq DNA IDs (NM) into their protein counterparts (if possible) using Perl-scripted calls. This step was necessary to accept the RefSeq identifiers into the GenMAPP gene database.

### Evaluation of Gene Ontology classification and pathway visualization

The Gene Ontology [53] vocabulary is widely used to classify genes of interest into functional categories. Furthermore, genes can also be interpreted as part of biological pathways; this allows both statistical and visual interpretation of gene expression data on a more meaningful level. Our aim here was to see whether our annotation approaches resulted in richer GO classifications and improved possibilities for pathway evaluation. We used GenMAPP 2.1 [11] for both the GO and the biological pathway analysis. For the Incyte array the original UniGene annotation was compared with the new annotation derived after combination of the two approaches. For each reporter on the Agilent G2519A Option 2 Mouse Development 44K the RefSeq DNA IDs (NM, RN and NX) were extracted from the most current Agilent annotation file to date (Annotation date: 1^st ^June 2005). These IDs were directly converted to their RefSeq protein (NP) counterparts using both the Mouse RefSeq database (release 20) and Perl-scripted calls. This was a necessary step because GenMAPP only accepts NP identifiers. For reporters with more than one RefSeq ID only the first reference was taken.

To see the actual improvement in gene visualization on biological pathways, both the Mm-Std_20060628 gene database and the "Mm_Contributed_20060920" MAPP-set were acquired using the GenMAPP 2.1 program [11]. The local Mouse MAPP-set consisted of 101 individual pathways, grouped by their function (physiological, metabolic, signaling and molecular). For reporters with more than one annotation only the first identifier was used, representing the highest quality hit. MAPPFinder [24] was used to keep track of the (total) amount of genes that can be displayed on these pathways. First, all reporters were classified in 4 classes (table 4), based on the possibility to connect the old and/or the new annotation to an entry in the GenMAPP gene database. For this classification we used the GenMAPP exception files. This classification was used to do the statistical evaluation in MAPPFinder and to visualize the results in GenMAPP (figures 5 and 6).

### Re-evaluation of old Incyte UniGene clusters towards UniProt identifiers

The GenMAPP program accepts UniGene cluster IDs as input for their gene database. Each UniGene cluster may contain protein similarity crosslinks (PROTSIM) towards multiple species including the native species. However, for each species these crosslinks only refer towards one single protein database: PIR, UniProt, RefSeq or PDB. UniGene entries often contain a separate Entrez Gene reference towards the native species. Based on the PROTSIM and EG crosslinks the UniGene cluster IDs can be converted to their UniProt counterpart, using a combination of different conversion tables derived from local copies of UniGene (Mouse build 151), LocusLink (050601) and the SwissProt database part in UniProt (Release 51). These crosslinks are first fully evaluated for the native species (mouse in our case) and next for other relevant species (here: rat and human). If a UniGene cluster contains a direct UniProt crosslink then that ID is kept. Next, a conversion table based on the LocusLink database transforms Entrez Gene references into UniProt IDs. After that, another conversion table based on crosslinks from the SwissProt part of UniProt towards PIR was used to convert PIR references in higher quality (SP) UniProt IDs. Finally references towards RefSeq were also converted using the conversion table based on Entrez Gene, thus making use of the relatively high curation quality of Entrez Gene. If no UniProt ID was found the whole procedure was repeated for other species of interest.

## Abbreviations

**BLAST **Basic Local Alignment Search Tool

**cEMBL **Cleaned EMBL

**ID **Identifier

**EC **Enzyme Commission number

**EG **Entrez Gene

**GO **Gene Ontology

**LL **LocusLink

**PDB **Protein Data Bank

**PIR **Protein Information Resource

**RS **RefSeq

**SP **SwissProt (UniProtKB/SwissProt)

**UG **UniGene

## Competing interests

The author(s) declares that there are no competing interests.

## Authors' contributions

SG designed the methods and provided a first draft of the manuscript. AvE and RvH provided both intellectual input for the manuscript and tested the methods. CE provided substantial advice and guidance during all phases of the project and assisted in the drafting of the manuscript. All authors have read and approved the final draft of the manuscript.

## Supplementary Material

Additional file 1 Comparison of Annotation Methods. This file contains tables that describe the outcome between our cEMBL method versus the RefSeq and TargetIdentifier approach. We randomly selected 1,000 reporters and compared their protein annotation wherever possible. We also counted the number of reporters that could be imported in GenMAPP using the approach-specific identifiers. Click here for file

Additional file 2 Local GenMAPP Pathway Ranking Results. This file contains the pathway ranking list, derived from visualizing either Agilent or Incyte reporter IDs to local pathways available in the GenMAPP program. These tables were cleaned out and sorted on pathway improvement ratio ("RefSeq vs New Ratio"). Click here for file
