# Supplementary material for: Linking microarray reporters with protein functions
Source: BMC Bioinformatics. 2007 Sep 26;8:360. doi: 10.1186/1471-2105-8-360 (PMC2140066; doi:10.1186/1471-2105-8-360)
Supplement: Additional file 1 — Comparison of Annotation Methods. This file contains tables that describe the outcome between our cEMBL method versus the RefSeq and TargetIdentifier approach. We randomly selected 1,000 reporters and compared their protein annotation wherever possible. We also counted the number of reporters that could be imported in GenMAPP using the approach-specific identifiers. [file 1471-2105-8-360-S1.doc]

**Extra Table 1 - Comparing our approach against RefSeq blasts**

*Our cEMBL/EnsEMBL based annotation was compared to the RefSeq approach for both the Incyte and Agilent arrays. For this purpose, one thousand reporters were randomly selected. The comparison of these approaches was done on both protein level and on number of reporters accepted in the GenMAPP Mouse Gene database (Mm-Std_20060628). The first two columns represent the meaningfulness of the annotation (protein or nucleotide). The next two columns represent the number of reporters that could be imported in the GenMAPP gene database using the resulting approach-specific identifiers.*

**
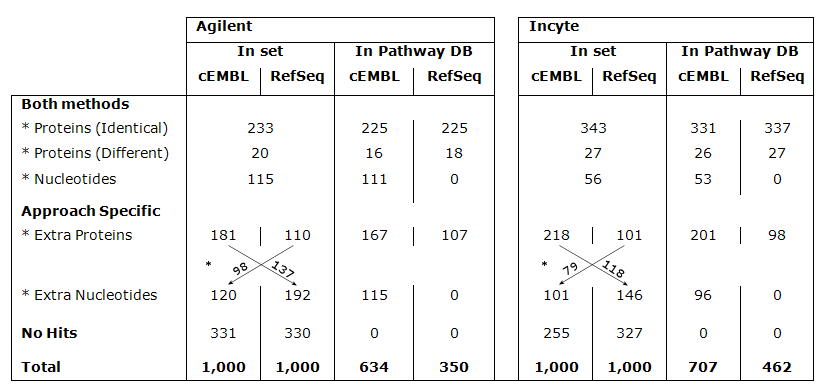
**

(*) The numbers on the lines represent the actual number of reporters that were annotated with a protein by one method, but with a nucleotide ID using the other method. Example: For Agilent the cEMBL approach linked 181 extra reporters to UniProt identifiers, of which 137 reporters were annotated by the RefSeq approach with a RefSeq DNA ID. Subtracting this number (137) from the total extra nucleotide IDs found by the RefSeq approach (192) results in finding 55 reporters annotated with a RefSeq DNA ID that were not found at all using the cEMBL approach. The same goes the other way around where cEMBL found 22 extra EnsEMBL gene identifiers.

**Extra Table 2 - Comparing our approach against the TargetIdentifier results**

*The annotation outcome of our combined cEMBL/EnsEMBL approach was compared to the TargetIdentifier (TI) method for the Incyte array on both protein level and acceptance in the GenMAPP Mouse Gene Database (Mm-Std_20060628). The same 1,000 random reporters of Extra Table 1 were used. The TargetIdentifier method returns only UniProt IDs, but often for other species then our main species of interest. The first two columns represent the meaningfulness of the annotation (protein or nucleotide). The next two columns represent the number of reporters that could be imported in the GenMAPP gene database using the resulting approach-specific identifiers.*

*
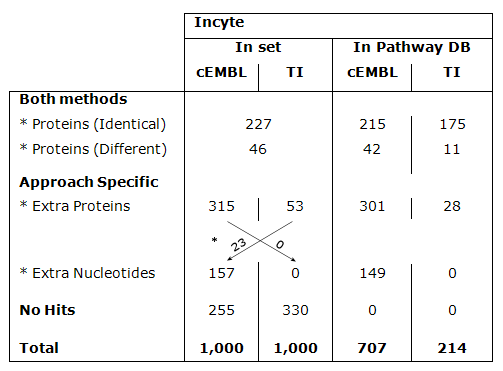
*

**(TI) TargetIdentifier**

**(*) See comment Extra Table 1**
